# Proctored adoption of robotic hiatus hernia surgery: outcomes and learning curves in a high-volume UK centre

**DOI:** 10.1007/s00464-023-10210-x

**Published:** 2023-07-20

**Authors:** Jennifer Straatman, Saqib A. Rahman, Nicholas C. Carter, Stuart J. Mercer, Benjamin C. Knight, Gijsbert I. van Boxel, Philip H. Pucher

**Affiliations:** Department of Upper Gastrointestinal Surgery, Portsmouth Hospitals University NHS Trust, Portsmouth, UK

**Keywords:** Hiatus hernia, Robotic surgery, Learning curve, Proctoring

## Abstract

**Background:**

The adoption of new surgical technologies is inevitably accompanied by a learning curve. With the increasing adoption of robotic techniques in benign foregut surgery, it is imperative to define optimal learning pathways, to ensure a clinically safe introduction of such a technique. The aim of this study was to assess the learning curve for robotic hiatal hernia repair with a pre-defined adoption process and proctoring.

**Methods:**

The learning curve was assessed in four surgeons in a high-volume tertiary referral centre, performing over a 100 hiatal hernia repairs annually. The robotic adoption process included simulation-based training and a multi-day wet lab-based course, followed by robotic operations proctored by robotic upper GI experts. CUSUM analysis was performed to assess changes in operating time in sequential cases.

**Results:**

Each surgeon (A, B, C and D) performed between 22 and 32 cases, including a total of 109 patients. Overall, 40 cases were identified as ‘complex’ (36.7%), including 16 revisional cases (16/109, 14.7%). With CUSUM analysis inflection points for operating time were seen after 7 (surgeon B) to 15 cases (surgeon B).

**Conclusion:**

The learning curve for robotic laparoscopic fundoplication may be as little as 7–15 cases in the setting of a clearly organized learning pathway with proctoring. By integrating these organized learning pathways learning curves may be shortened, ensuring patient safety, preventing detrimental outcomes due to longer learning curves, and accelerating adoption and integration of novel surgical techniques.

Minimally invasive techniques have been widely adopted in gastro-intestinal surgery, and have markedly improved patient outcomes in a wide array of procedures [1–3]. Robotic surgery, representing the latest advancement in surgical technology, is increasingly being adopted and has been associated with improved recovery rates for patients and better ergonomics for surgeons in colorectal and urological procedures [4, 5].

The advantages of 3D visualisation, elimination of the fulcrum effect, expanded articulation and better ergonomic positioning have led to increasing adoption of robotic platforms in upper gastro-intestinal (UGI), or foregut, surgery [6, 7], particularly in the United States. The more gradual adoption in Europe is currently accelerating [8].

Hiatal surgery (hiatus hernia repair and fundoplication) represents a key procedure for robotic benign UGI surgery, requiring numerous complex skills including tissue manipulation, hiatal dissection, and intracorporeal suturing. Mastering these skills is one of the reasons that mastery of benign hiatal robotic surgery is increasingly pursued as a “stepping stone” for surgeons adopting robotic UGI cancer surgery, as well [9].

Understanding the duration of any surgical learning curve, and any associated impact on patient outcomes, during the adoption phase of any novel procedure or approach, is crucial to ensuring ethical and safe introduction of new technologies into surgical practice. In the past, new surgical approaches were adopted without formalised training pathways, leading to a potential negative impact on patient outcomes [9–11]; it is now increasingly recognised that training curricula and proctored adoption may be crucial in ameliorating the learning curve [12]. Whilst published reports have examined learning curves for robotic surgery in other procedures or specialties, there are no published reports for fundoplication/hiatus hernia repair, despite the complexity of this procedure, and “stepping stone” status in some curricula for surgeons adopting robotic oesophagectomy.

Here we present the learning curve for robotic fundoplication with a defined adoption process and proctoring.

## Materials and methods

A retrospective analysis was performed of a prospectively maintained database in our hospital, a high-volume referral center for hiatal hernia surgery. Following institutional approval, all robotic hiatus hernia repairs from February 2019 up to February 2022 inclusive were identified from a prospectively maintained dataset of patients. This period captures the initial adoption phase for robotic surgery in our department, a high-volume laparoscopically experienced tertiary upper GI unit performing over 100 hiatus hernia repairs per year, offering both upper GI cancer and bariatric services, in the South of England. The design and analysis was performed in line with the STROBE guideline for observational studies [13]. Informed consent was waived due to the observational nature of the study.

### Surgical approach

The robotic approach was performed in an identical manner as the previously mastered laparoscopic approach, using the DaVinci X robotic platform (Intuitive Surgical, Sunnyvale, CA, USA); later procedures utilised the DaVinci Xi, these took place beyond the point of the learning curve. Circumferential hiatal dissection and mobilisation was performed using hook diathermy to reduce the hiatus hernia and ensure adequate intra-abdominal length. A posterior crural repair was performed with interrupted permanent sutures. All fundoplications were performed in a standardised fashion using an anterior 180-degree wrap, securing the fundus to left and right crura with interrupted sutures; no oesophageal lengthening procedures were performed.

### Robotic surgery adoption process

The adoption process included virtual reality simulation-based training, followed by a multi-day wet lab-based course involving animal and cadaver operating. Following completion of this training scheme, robotic surgery was commenced with sequential hiatal surgery cases, proctored by experienced, accredited, and internationally recognised trainers in robotic upper GI surgery. These trainers are accredited by industry based on extensive expertise and experience in the surgical procedures and approach in question. Additional surgical time was allowed for training during the learning curve period, until surgeons were formally signed off for independent practice by the proctors.

Three surgeons who started with robotic surgery during the study period (surgeons A, B, C) were previously naïve to robotic surgery and went through the adoption process as described above; a fourth surgeon (surgeon D) appointed to the hospital at the start of the study period had already established their robotic competency elsewhere and as such acted as a control for the learning curve analysis as it was anticipated that they would have already overcome their learning phase. Surgeons A/B/C all had extensive experience with at least 10 years independent practice each and having performed over 100 fundoplications each.

Not all surgeons in the unit adopted robotic surgery during the study period and as such the number of patients included in this study is less than overall unit volume. For the surgeons who did adopt robotic surgery (surgeons A, B, C), all hiatus hernia repairs during the adoption phase were performed robotically and as such these represent consecutive cases for each surgeon. There was no change in patient referral process, surgeons thus saw and operated upon patients as they would have in standard fashion (i.e. without obvious selection bias).

### CUSUM analysis

Cumulative sum (CUSUM) analysis was performed to assess the learning curve of robotic anti-reflux surgery. Due to the low rate of adverse perioperative outcomes in this patient group, operating time was used as a surrogate of acquisition of skill, as is typical for such an analysis, rather than clinical outcomes. Operating time was defined as the total time between the first surgical incision and skin closure and includes robot docking time.

To perform the CUSUM analysis/plot, the observed operating time for each consecutive case for each surgeon was compared to the cumulative mean ‘expected’ time, with the difference cumulatively added (summed) to the preceding value. Thus, if a learning curve exists, the observed time exceeds the mean time the CUSUM increases (and the trace trends upwards) and conversely if the observed time was less than the mean time the CUSUM decreases, representing an overall reduction in mean operating time as the learning curve is overcome and operating times start to reduce. CUSUM plots were generated for each individual surgeon.

The study period also contained a number of more complex hiatal procedures, which would be expected to have a longer operating time. The following procedures were categorised as complex cases; revisional cases following previous hiatal hernia repair, incarcerated hiatus hernia, and giant hiatus hernia with either a total intrathoracic stomach, or herniation of abdominal viscera other than the stomach, or both. These complex procedures were identified from the group using procedure coding and surgical notes.

These cases were included in the plot using a risk adjusted cumulative sum analysis (RA-CUSUM). Here, the expected time is calculated from a surgeon-specific linear regression model with operating time as the independent variable and operation complexity (yes/no) and operation number as the dependent (predictor) variables. The resulting mean difference in operating times between simple and complex cases based on the regression model was used to adjust for case complexity when including both in the CUSUM analysis.

A visual assessment of inflection point was used to judge the point at which the learning curve had been overcome and mean operating times began to reduce.

### Statistical analysis

Hypothesis testing was performed using non-parametric statistics including the Mann–Whitney U test and Chi-square test, with CUSUM assessed graphically. A two-tailed *p* value < 0.05 was considered statistically significant. Analysis was conducted in R 4.4.1. With regard to sample size, we assessed correction to be applied for standard and advanced cases, indicating at least 20 patients had to be included per surgeon.

## Results

In total, 109 cases were included in the study. Each surgeon performed between 22 and 32 cases over the study period. Overall, 40 cases were identified as ‘complex’ (36.7%), including 16 revisional cases (14.7%), with a larger proportion of complex cases performed by surgeons B and C. Median operating time was 113 min, 52/109 (47.7%) of cases were performed as a day-case and the median length of stay was 1 day. Among non-complex (primary, type 1 hiatus hernia) operations, the median operating time was 104 min. Only one case was converted to a laparoscopic approach, due to difficult visualisation and dissection. No intra-operative complications occurred. Case characteristics stratified by surgeon are summarised in Table 1.

**Table 1:** Patient and operative characteristics

Parameter	Overall	Surgeon A	Surgeon B	Surgeon C	Surgeon D	*p*-value
N	109	22	32	31	24	
Male Gender	43 (39.4)	9 (40.9)	11 (34.4)	15 (48.4)	8 (33.3)	0.61
Age	56.0 [46.6–71.3]	69.8 [50.4–76.3]	50.4 [38.6–58.3]	61.1 [53.5–72.9]	55.4 [47.2–70.3]	0.009*
BMI	30.0 [26.0–33.0]	30.0 [27.0–33.5]	31.5 [27.0–33.8]	26.5 [23.0–30.2]	30.0 [26.5–31.8]	0.111
Hiatus hernia length	4.0 [3.0–5.8]	4.0 [3.0–4.8]	4.0 [3.0–5.0]	4.0 [3.0–6.0]	5.0 [4.0–6.2]	0.243
Revision/Reoperation	15 (13.8)	1 (4.5)	0 (0.0)	10 (32.3)	4 (16.7)	0.001*
Complex Case	40 (36.7)	3 (13.6)	5 (15.6)	21 (67.7)	11 (45.8)	< 0.001*
Surgical Time (minutes)	113.0 [95.0–139.0]	111.5 [94.5–129.0]	105.5 [88.0–127.2]	121.0 [96.5–142.0]	123.5 [105.0–149.2]	0.307
Length of Stay (days)	1.0 [0.0–1.0]	0.5 [0.0–1.0]	0.0 [0.0–1.0]	1.0 [0.5–2.0]	1.0 [0.0–1.2]	0.01*
Day case procedures	52 (47.7)	11 (50.0)	23 (71.9)	8 (25.8)	10 (41.7)	0.003*

Data Presented as median [IQR] and absolute count (%). Chi-square test, except Mann Whitney U Test, *p < 0.05

Two major post-operative complications (Clavien–Dindo score >  = 3) were recorded, one patient returned to the operating theatre for partial release of cruroplasty sutures for post-operative dysphagia one week after primary surgery, another suffered small bowel obstruction through a port-site hernia (8 mm robotic trocar entry site).

Median follow-up was 15 months (range 4–37 months). Of the patients undergoing surgery during the study period, two (2/109, 1.8%) had a further elective procedure for recurrent symptoms within the follow-up period: one underwent revisional surgery for recurrence following a primary hiatus hernia repair, another for repeated recurrence following revisional surgery.

### CUSUM analysis

**Fig. 1:**
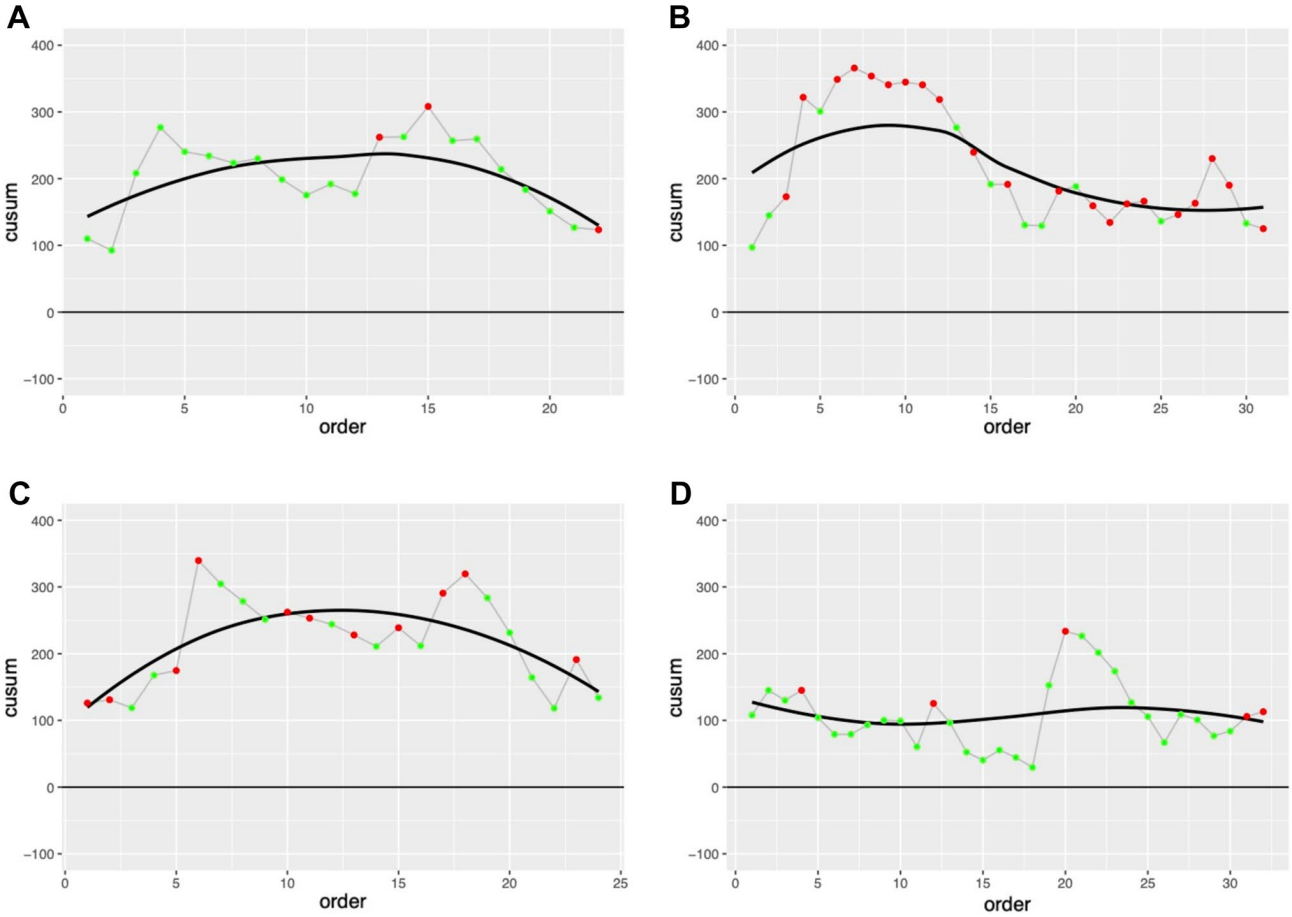
Unadjusted CUSUM analysis for each surgeon. Green dots reflect non ‘complex’ cases and Red dots ‘complex’ cases. A fitted LOESS curve is plotted to the absolute trends

**Fig. 2:**
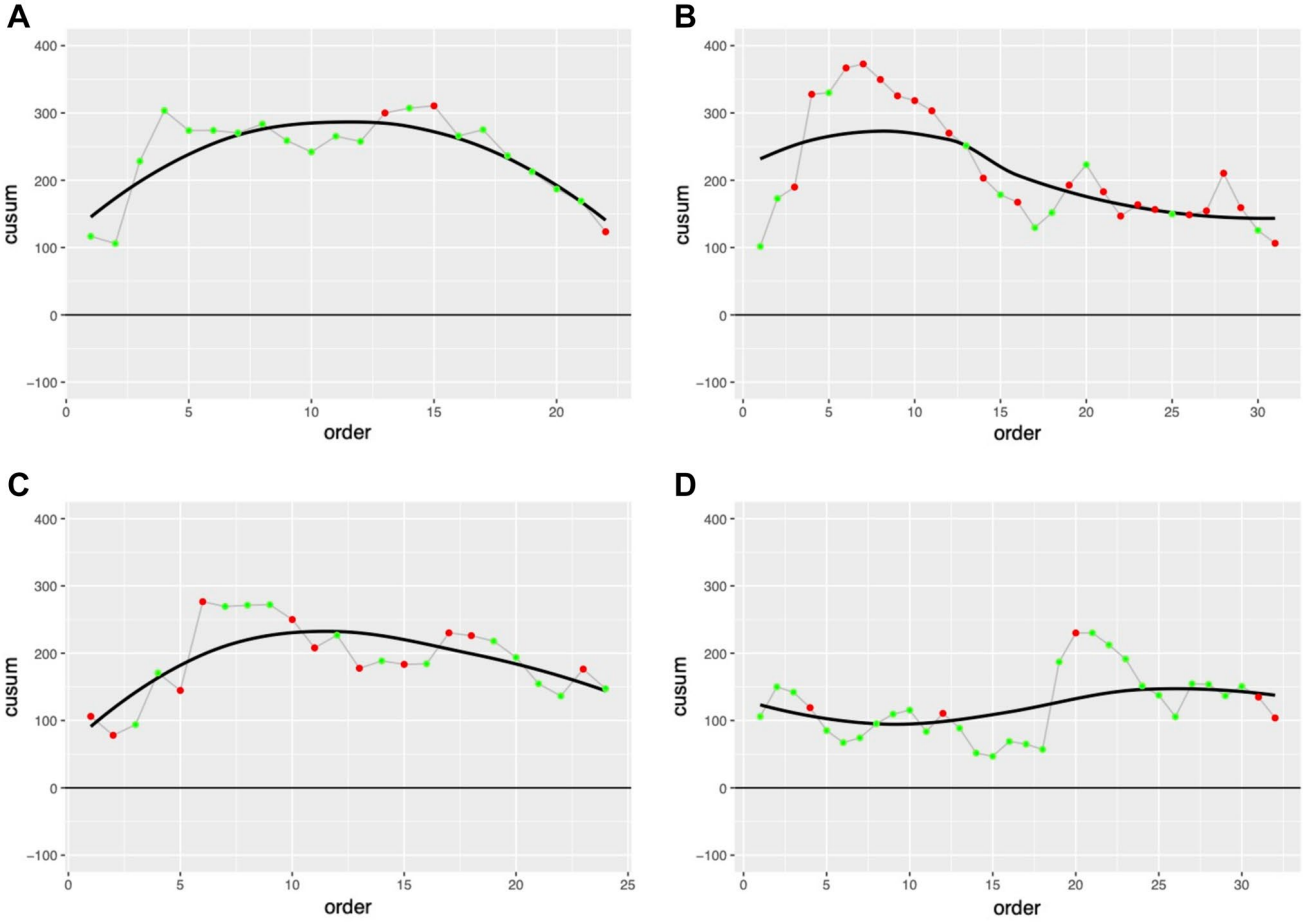
RA-CUSUM analysis (adjusted for case complexity and order) for each surgeon. Green dots reflect non ‘complex’ cases and Red dots ‘complex’ cases. A fitted LOESS curve is plotted to the absolute trends

Unadjusted CUSUM analysis is presented for reference in Fig. 1. Following adjustment of complex cases using a linear regression model as seen in Fig. 2, visual inflection points are seen after 7 (surgeon B) to 15 cases (surgeon A). Surgeon D, already competent in robotic surgery, maintained a flat CUSUM curve with no appreciable inflection point.

## Discussion

The adoption of new surgical technologies is inevitably accompanied by a learning curve. The role of modern health systems must be to transfer medical and surgical advances to their patients in a manner that is clinically safe (equivocal or superior outcomes, even during the learning curve), fiscally responsible (minimal wasted resources, and keeping the learning curve as short as possible where this slows down surgical efficiency), and transparent to the patient and organisation.

This study suggests that such an adoption process allows safe and rapid skill accrual, with initial skill plateaus as judged by CUSUM inflection points achieved in as little as 7–15 cases. Although, it should be noted, the here presented study is not a comparative study, it does reflect a short learning curve for robotic fundoplication in a proctored setting, in surgeons with extensive experience in laparoscopic foregut surgery.

A recent study on learning curve for robotic hiatal hernia repair and fundoplication reported a learning curve of 40 cases and mastery was achieved after 85 cases [14]. The cases were non-proctored. Another study on learning curves for robotic foregut surgery suggested a long learning curve of up to 86 cases, although it should be noted this study included multiple types of foregut surgery [15]. Similar results hold for the learning curve for conventional laparoscopic fundoplication; studies state that the learning curve ranges from 20 to 50 cases, and improvements are seen even beyond 400 cases [16–18]. Going through the learning curve for minimally invasive fundoplication can have detrimental effects for patients, with some studies reporting a higher conversion rate during the learning curve [19], this further stresses the need for an optimization of learning pathways, aiming to diminish learning curves.

The implications of the here presented study are manifold; firstly, that through a formalised adoption process including a simulation-based curriculum and expert proctoring, that the learning curve for robotic fundoplication surgery can be abbreviated and delivered in a safe fashion. Second, that through such a process the incorporation of new surgical technology can be more quickly rolled out through an entire unit with multiple surgeons; reducing costs, promoting equity, and advancing surgical practice, especially when compared to more traditional models of a single surgeon slowly learning independently. Finally, we contribute objective data on learning curves for robotic fundoplication, utilising a novel method of regression-based casemix adjustment of CUSUM calculation, which may serve to benchmark future studies. There is a dearth of guidance for the adoption of robotic upper gastro-intestinal surgery [12], with only nascent efforts being developed in other specialties such as colorectal surgery and urology [20, 21].

The benefits of formalised systems of coaching and proctoring are well recognised in surgery [22]; previous studies have reported the effect of the trainer on the learning curve for surgical procedures, wherein detailed analysis of individual learning curves for different pupils revealed that the trainer was the most important factor influencing the performance score [23]. In one study, the absence of experienced help was found to be an individual factor associated with failure (conversion or early reoperation) in the learning curve for laparoscopic fundoplication [17]. Similar results with regards to the effect of training and proctoring on the learning were observed for transthoracic robotic minimally invasive esophagectomy; where a shorter learning curve was observed when a structured pathway, which included proctoring, was adopted [24]. Increasingly, robotic surgery platform manufacturers are hosting courses to teach trainee surgeons basic skills in robotics, and implementation of these teachings in a surgical curriculum may aid in early proficiency in basic procedures [25]. As is the case in other specialties wherein robotic surgery is already established, more specialized fellowships, such as the UGIRA (Upper GI International Robotic Association) fellowship may further aid in setting up young surgeons to go through their learning curve in an organized, proctored manner.

In addition to the adoption process, learning curves are also dependent on the complexity of the procedure and prior surgeon experience. While laparoscopic fundoplication is considered a complex laparoscopic foregut procedure and requires adequate skills for dissection and suturing, it is also considered a stepping stone to more advanced procedures, such as large hiatal hernia repairs, revisional surgery and oesophagogastric resections [26]. Learning curves for laparoscopic Roux-en-Y gastric bypass, for example, have been shown to be shorter in surgeons who were already experienced in other laparoscopic techniques [27]. Similar results may be reasonably assumed for robotic assisted surgery, and for the acquisition of skills in robotic surgery a step-up approach in procedural complexity could be beneficial to further shorten learning curves.

The effect, and duration, of learning curves may also impact upon surgical quality control, and affect outcomes, dissemination, and further uptake of robotic surgery. To date, for example, only four randomised trials have compared laparoscopic to robotic fundoplication. Outcomes of these trials, now technologically outdated with most recent trial now over 15 years old, did not show differences in outcomes for robotic versus conventional laparoscopic fundoplication [28–31], with regard to length of hospital stay, post-operative complications and patient symptoms scores. However, surgeons’ experience was mentioned in only one study with minimal prior experience [28], whereas none of the studies reported on training and/or adoption pathways with reference to robotic surgery.

This study reflects the experience of a single centre. Surgeons A–C all had significant experience, having been in independent practice for 8–15 years, with shared practices within the department meaning that all had roughly equivalent experience and techniques. Despite this, assessment of the patient cohorts revealed differences in casemix per surgeon, which is likely to reflect some degree of selection bias, with surgeon B performing a disproportionate number of complex cases—but also demonstrating the shortest learning curve. The study period also included two periods during which all benign surgical activity was halted due to the COVID-19 pandemic, though these periods occurred in the latter half of the study where the initial inflection points for surgeons were overcome. It is possible that reduced operative activity during this early phase of robotic surgery may have flattened the curve and that with ongoing normal activity the inflection point of the CUSUM curve might have been steeper still. Finally, despite some heterogeneity of cases and case complexity, and the fact that this is well recognised limitation of learning curve analyses in general, we utilised a novel approach to adjust for more complex variants of the procedure being studied, with a regression model adjustment which we suggest increases the validity of our findings.

Robotic fundoplication is considered feasible and safe, based on currently available literature [32, 33], and robotic fundoplication is now fully embedded in our hospital. In the future we aim to utilise standardised learning pathways, such as the proctored pathway presented here, and implement them in larger training programs. Safe learning of low complexity robotic upper GI surgery, may further aid in attenuating learning curves for more complex cases and procedures, such as robotic oesophagectomy.

## Conclusion

The learning curve for robotic fundoplication may be as little as 7–15 cases in the setting of a clearly organized learning pathway with proctoring. By integrating these organized learning pathways learning curves may be shortened, ensuring patient safety, preventing detrimental outcomes due to longer learning curves, and accelerating adoption and integration of novel surgical techniques.
